# Isolation and Expansion of Multipotent Progenitors from Human Trabecular Meshwork

**DOI:** 10.1038/s41598-018-21098-2

**Published:** 2018-02-12

**Authors:** Yuan Zhang, Subo Cai, Scheffer C. G. Tseng, Ying-Ting Zhu

**Affiliations:** 1 Research and Development Department, Tissue Tech, Inc, Miami, FL USA; 2 Department of Ophthalmology, Union Hospital, Tongji Medical College, Huazhong University of Science and Technology, Wuhan, China

## Abstract

To expand multi-potent progenitors from human trabecular meshwork (TM), we have created a new optimized method on two-dimensional (2D) followed by three–dimensional (3D) Matrigel in modified embryonic stem cell medium supplemented with 5% fetal bovine serum (MESCM + 5% FBS). The expanded TM cells were small cuboidal cells expressing TM markers such as AQP1, MGP, CHI3L1, and AnkG, embryonic stem cell (ESC) markers such as Oct4, Sox2, Nanog, and ABCG2, and neural crest (NC) markers such as p75NTR, FOXD3, Sox9, Sox10, and MSX1. Although expanded cells lost expression of these markers after passage, the cells regained the markers when Passage 2 cells were seeded on 3D Matrigel through activation of canonical BMP signaling. Such restored progenitors could differentiate into corneal endothelial cells, adipocytes, and chondrocytes but not keratocytes or osteocytes. Therefore, we have concluded that human TM harbors multipotent progenitors that can be effectively isolated and expanded using 2D Matrigel in MESCM + 5% FBS. This unique *in vitro* model system can be used to understand how TM is altered in glaucoma and whether such TM progenitor cells might one day be used for treating glaucoma or corneal endothelial dysfunction.

## Introduction

The main outflow pathway for aqueous humor in the eye consists of a series of endothelial cell–lined channels in the angle of the anterior chamber comprising the trabecular meshwork (TM), Schlemm’s canal, the collector channels, and the episcleral venous system. The TM, especially the juxtacanalicular region and the inner wall of Schlemm’s canal are thought to be the source of the most resistance to aqueous outflow. Reduced cellularity and function within the TM is observed with age and correlates with increased outflow resistance and elevated intraocular pressure (IOP)^1–3^. Consequently, dysfunction of TM cells might play a role in blindness caused by glaucoma^4^.

As a first step to explore the pathogenic role of TM cells in glaucoma, it is necessary to isolate and expand TM cells *in vitro*. In this regard, cumulative evidence suggests that there is a population of progenitors in Schwalbe’s Ring, which is the transition area between the periphery of the corneal endothelium and the anterior non-filtering portion of the TM^5^. This hypothesis primarily comes from the following observations: (1) an increase in TM cell division is localized to the anterior non-filtering portion of the TM after argon laser trabeculoplasty^6^ and (2) stem cell markers such as nestin, alkaline phosphatase and telomerase are expressed in cells in the transition region^7^. In addition, Du *et al*.^8,9^ have isolated human TM cells by collagenase digestion and cultured on plastic in stem cell growth medium (SCGM) and such cells expressed ABCG2, Notch1, Oct4 and MUC1. Primarily isolated and expanded side population of TM cells can then differentiate into keratocytes and expanded TM cells can home to mouse TM and differentiate into TM cells *in vivo*^9^, suggesting that these cells might be progenitor cells.

Herein, we report our effort in optimizing the method of expanding TM progenitor cells using 2D Matrigel and MESCM + 5% FBS. Our method yields small cuboidal TM progenitors expressing markers of TM, embryonic stem (ES), and NC cells. Unlike what has been reported by Du *et al*.^8^, these progenitor cells can be differentiated into corneal endothelial cells, adipocytes, and chondrocytes but not keratocytes or osteocytes. Interestingly, maintenance of such a progenitor status requires activation of the canonical BMP signaling. The significance of these findings in studying the pathogenesis and being deployed as a cell-based therapy for glaucoma and corneal endothelial dysfunction is further discussed below.

## Results

### 2D Matrigel is preferred to maintain TM morphology and phenotype

**Figure 1:**
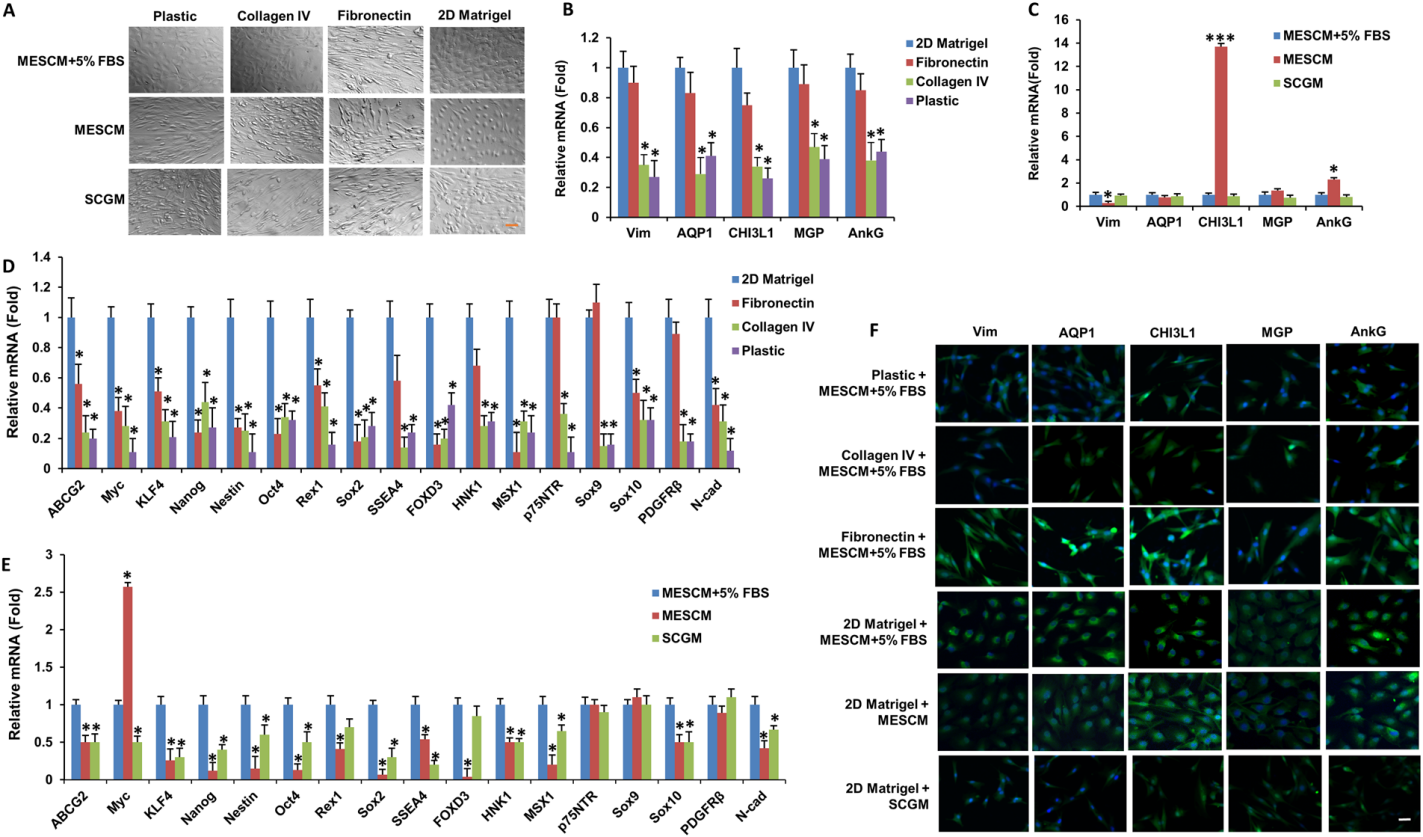
2D Matrigel is preferred to maintain TM morphology and phenotype. Freshly isolated TM cells were cultured on four different substrates and three different media for 4 weeks before subjected to morphological analysis by phase-contrast microscopy (**A**, scale bar: 100 µm), transcription expression of TM markers (**B**, **C**) and ESC and neural crest markers (**D**,**E**, using cells cultured on 2D Matrigel in MESCM + 5% FBS as the control, n = 3, *P < 0.05 and ***P < 0.001) and immunostaining of TM markers (**F**, scale bar: 20 µm).

A number of substrates such as fibronectin, gelatin (collagen) and plastic without coating have been used to expand TM cells^8–15^. A number of media have also been used to culture TM cells including Dulbecco’s modified Eagle’s Medium (DMEM) + 10% FBS^12,14,15^, Medium 199E + 20% FBS^10,11^, Improved Minimum Essential Medium (IMEM) + 10–20% FBS^13^ and SCGM (a stem cell culture medium with 5% FBS)^8,9^. Because we have successful expanded human limbal niche cells on 2D Matrigel in MESCM^16–18^, we thus compared four substrates, i.e., plastic, fibronectin, collagen IV, and 2D Matrigel and three media, i.e., MESCM, MESCM + 5% FBS, and SCGM. When cultured in the same medium, i.e., MESCM + 5% FBS, freshly isolated TM cells turned to “spindle” fibroblastic-like cells after 4 weeks of culture on plastic, fibronectin, and collagen IV but remained cuboidal on 2D Matrigel (Fig. 1A, top panel). Under this culturing condition, the transcript expression of TM markers (such as AQP1, CHI3L1, MGP, and AnkG) was maintained when cultured on 2D Matrigel or fibronectin but significantly reduced on plastic or collagen IV (Fig. 1B), and the immunostaining data also showed the same result (Fig. 1F, top four panel). However, the transcript expression of ESC and NC markers by cells cultured on 2D Matrigel was significantly higher than those cultured on fibronectin, plastic, or collagen IV (Fig. 1D). These results suggest that 2D Matrigel was the preferred substrate to maintain TM morphology and phenotype when cultured in MESCM + 5% FBS.

When cells cultured on 2D Matrigel was changed from MESCM + 5% FBS to SCGM, they also turned into spindle cells (Fig. 1A, bottom panel). Cells remained somewhat cuboidal in MESCM without 5% FBS (Fig. 1A, middle panel) and had similar transcript expression of TM cell markers (such as AQP1, MGP and stromal marker Vim) but significantly higher transcription expression of CHI3L1 and AnkG (Fig. 1C), which was confirmed by immunostaining (Fig. 1F). However, the cell’s transcript expression of ESC and NC markers such as ABCG2, KLF4, Nanog, Nestin, Oct4, Rex1, Sox2, SSEA4, FOXD3, HNK1, MSX1, Sox10 and N-cadherin was significantly less than those cultured in MESCM + 5% FBS (Fig. 1E).

### MESCM + 5% FBS is preferred to promote expansion

**Figure 2:**
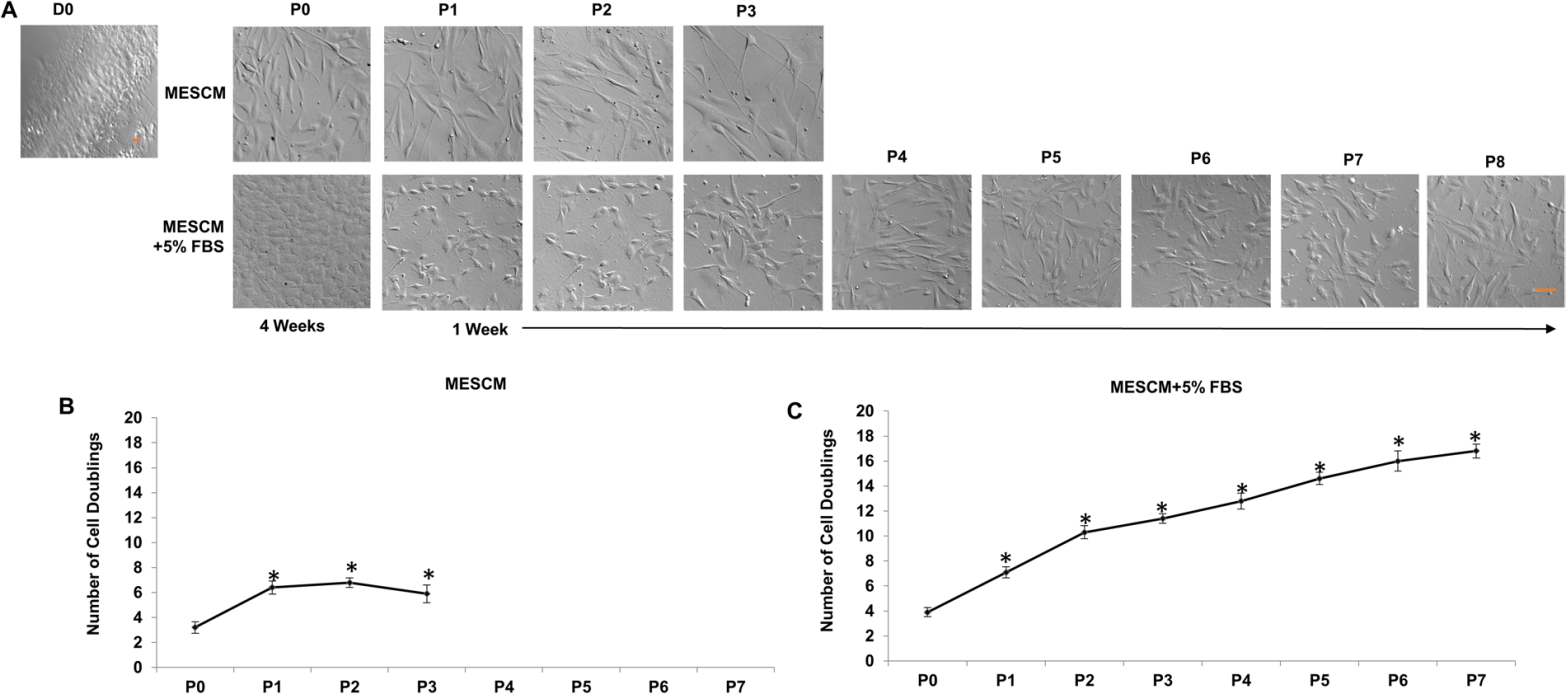
MESCM + 5% FBS is preferred to promote expansion. TM cells were expanded on 2D Matrigel in MESCM or MESCM + 5% FBS and each passage was subjected to morphological analysis by phase-contrast microscopy (**A**, scale bar: 100 µm). Cells expanded in MESCM alone (**B**) yielded fewer passages and total cell doubling than those expanded in MESCM + 5% FBS (**C**, n = 3. *P < 0.05 when compared to that of P0 cells).

Although both MESCM and MESCM + 5% FBS were able to maintain the TM phenotype in freshly isolated TM cells (Fig. 1), TM cells cultured in MESCM without FBS did not grow well at P1 and stopped growing at P3 with an enlarged shape and cell debris (Fig. 2A, top panel, and Fig. S1), resulting in a total cell doubling of 7 (Fig. 2B). In contrast, TM cells cultured in MESCM + 5% FBS could be expanded to 8 passages with a total cell doubling of 16 (Fig. 2C). Hence, we concluded that expansion of TM cells was promoted in MESCM + 5% FBS, but not MESCM, when seeded on 2D Matrigel.

**Figure 3:**
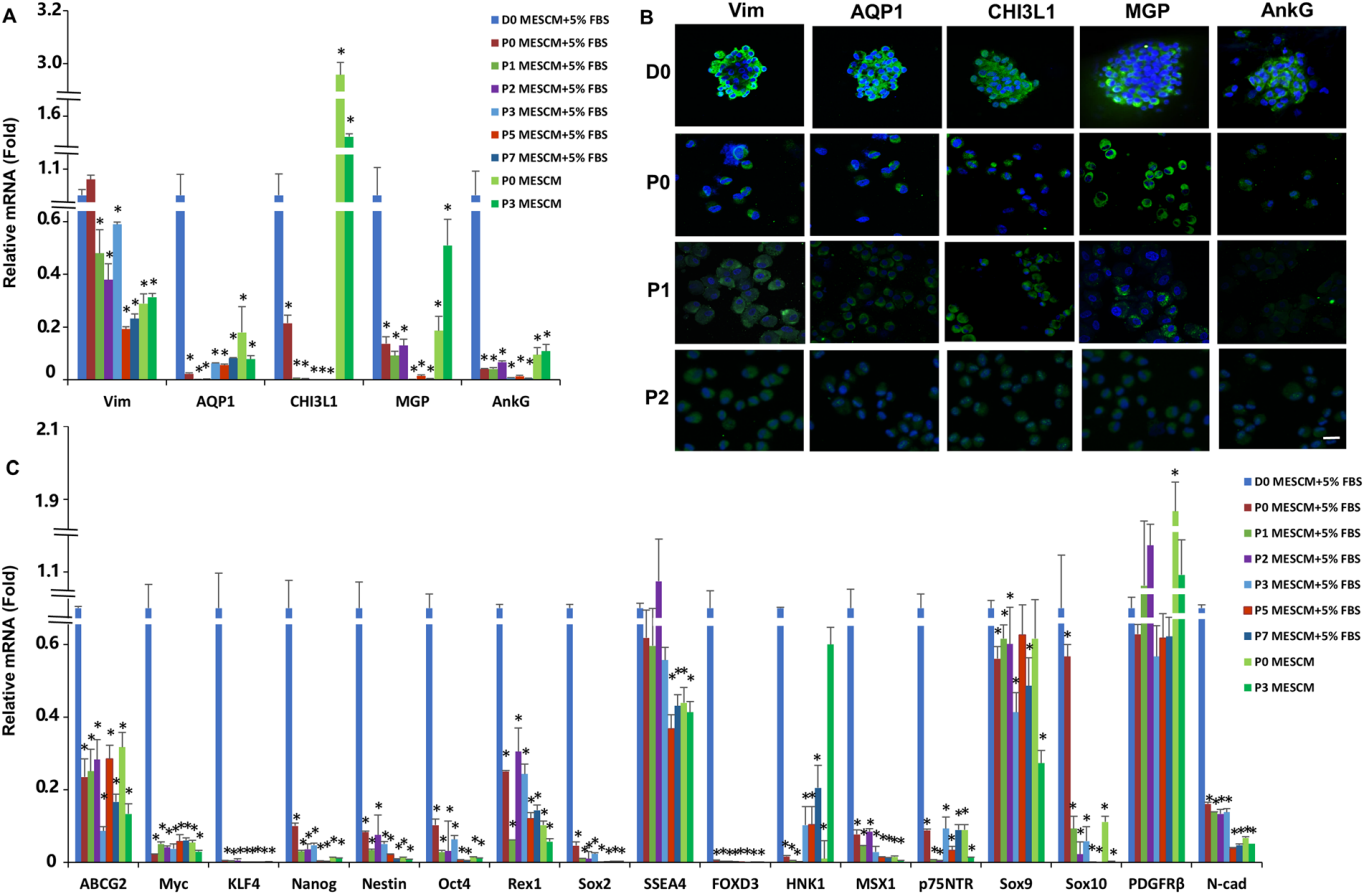
Expanded TM cells lose their phenotype and progenitor status on 2D Matrigel. Freshly isolated TM cells (D0) and TM cells cultured on 2D Matrigel in MESCM + 5% FBS were subjected to qRT-PCR for transcription expression of TM markers (**A**) and ESC and neural crest markers (**C**, using freshly isolated TM cells as the control, n = 3, *P < 0.05) and immunostaining of TM markers (**B**, scale bars: 20 µm).

However, there was a gradual loss of the TM phenotype during serial passages on 2D Matrigel in MESCM + 5% FBS as evidenced by significant reduction of transcript expression of TM markers such as AQP1, CHI3L1, MGP, AnkG and the stromal marker Vim (Fig. 3A). Immunostaining showed that freshly isolated TM cells (D0) positively expressed all these four markers (Fig. 3B) similar to what have been previously reported^9,19^. However, TM cells exhibited reduced expression of MGP and AnkG more so than CHI3L1 and AQP1 after Passage 1 (Fig. 3B). In addition, the loss of the TM phenotype was also accompanied by significant reduction of transcript expression of ESC markers such as ABCG2, Myc, KLF4, Nanog, Oct4, Rex1, and Sox2 as well as NC markers such as Nestin, FOXD3, HNK1, MSX1, p75NTR, Sox9, Sox10 and N-cadherin (Fig. 3C). Collectively, these results suggested that 2D Matrigel in MESCM + 5% FBS could not maintain the TM phenotype although it promoted cell expansion.

Because Du *et al*. have reported that CD73, CD90, CD166 and Bmi1 are valuable stem cell markers^8^, we have also performed their expression in TM cells at different culture stages. Although CD90 is a multipotent stem cell marker for the other model system^8^, its transcript has not been found in TM cells in our system. Though the transcripts of CD73, CD166 or Bmi1 have been found in TM cells expanded in our system, CD73 is upregulated from passage 1 to passage 7, CD166 is downregulated from passage 2 to passage 7, and Bmi1 is upregulated at passage 1 (Supplemental Fig. 2A). In addition, the expression of those markers is not affected by 3D Matrigel (Supplemental Fig. 2B).

### TM phenotype and progenitor status is reversed by 3D Matrigel

**Figure 4:**
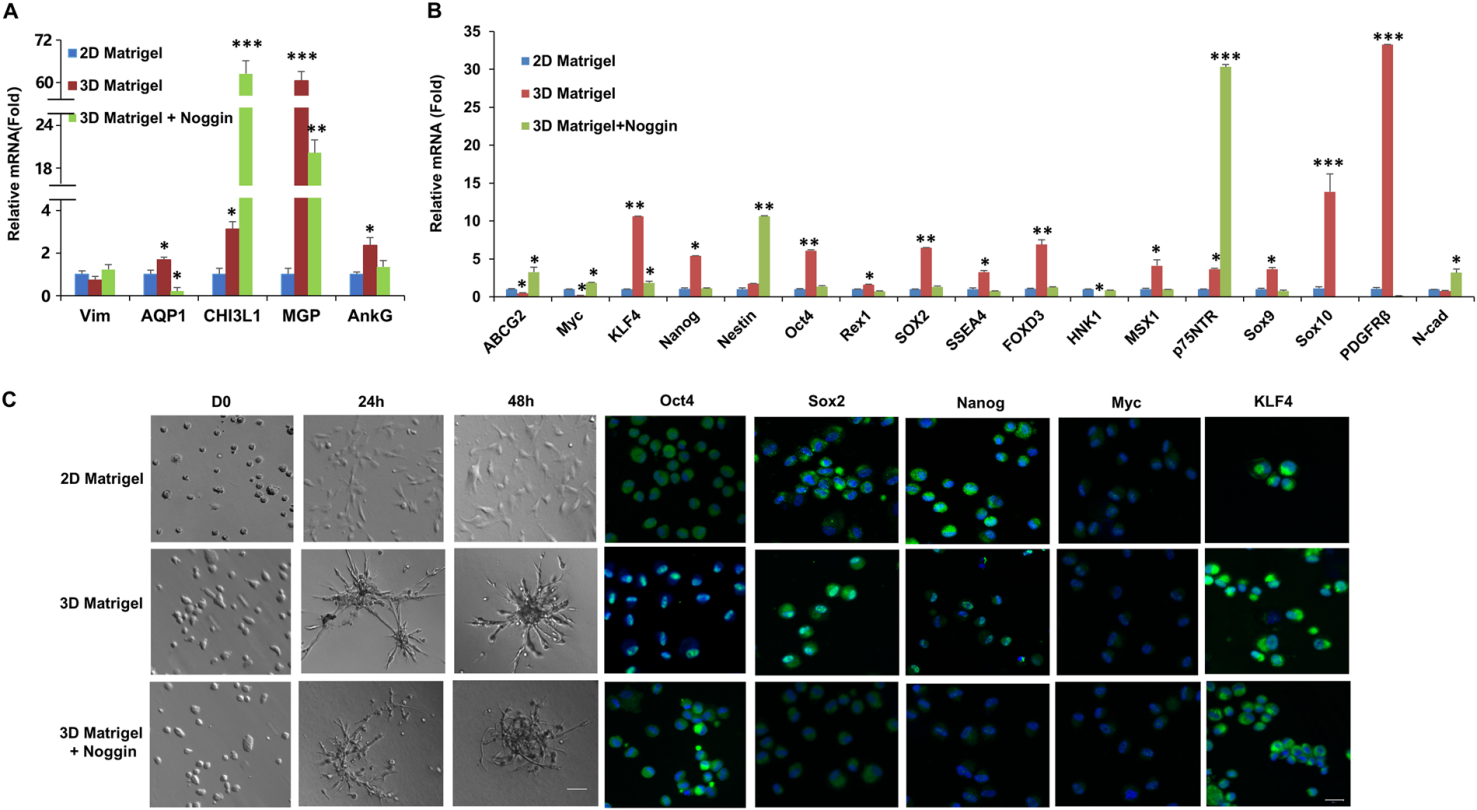
3D Matrigel promotes aggregation and upregulates expression of markers of TM cells, ESCs and NC. P3 cells cultured on 2D Matrigel in MESCM + 5% FBS were reseeded in 3D Matrigel with or without Noggin before morphological analysis by phase contrast microscopy (**C**, scale bar: 20 µm), immunostaining to Oct4, Sox2, Nanog, Myc, or KLF4 (**C**, nuclear counterstained by Hoechst 33342, scale bar: 20 µm), and qRT-PCR for TM markers (**A**) and ESC and neural crest markers (**B**, n = 3, **P < 0.01 and ***P < 0.001) by setting the expression level for 2D Matrigel as the control.

The gradual loss of the *in vivo* phenotype on 2D Matrigel has also been noted during expansion of human limbal niche cells and such a loss can be reversed by reseeding cells on 3D Matrigel^17,18,20^. To test this, we passaged 2 × 10^4^ per cm^2^ of P2 TM cells on 2D Matrigel as a control and 3D Matrigel with or without Noggin in MESCM + 5% FBS. Upon reseeding back to 3D Matrigel for 48 h, these cells formed spheres (Fig. 4C). RT-PCR disclosed significant upregulation of the transcript level of TM cell markers such as AQP1, CHI3L1, MGP and AnkG except stromal marker vimentin (Vim) (Fig. 4A) as well as embryonic stem cell (ESC) and NC markers such as KLF4, Nanog, Oct4, Sox2, SSEA4, FOXD3, MSX1, Sox9, Sox10 and PDGFRβ when compared to that of P3 cells still cultured on 2D Matrigel in the same medium (Fig. 4B). Such upregulation of TM, ESC and NC markers except TM marker CHI3L1, ABCG2, Myc, Nestin, p75NTR and N-cadherin was attenuated by addition of Noggin (Fig. 4B and C). Immunostaining showed nuclear translocation of Oct4, Sox2, Nanog and KLF4 in TM cells cultured on 3D Matrigel, but not in cells seeded on 2D Matrigel (Fig. 4C). Addition of Noggin abolished nuclear translocation of Oct4, Sox2, Nanog and KLF4 in cells seeded on 3D Matrigel (Fig. 4C).

### BMP signaling is activated on 3D Matrigel Culture

**Figure 5:**
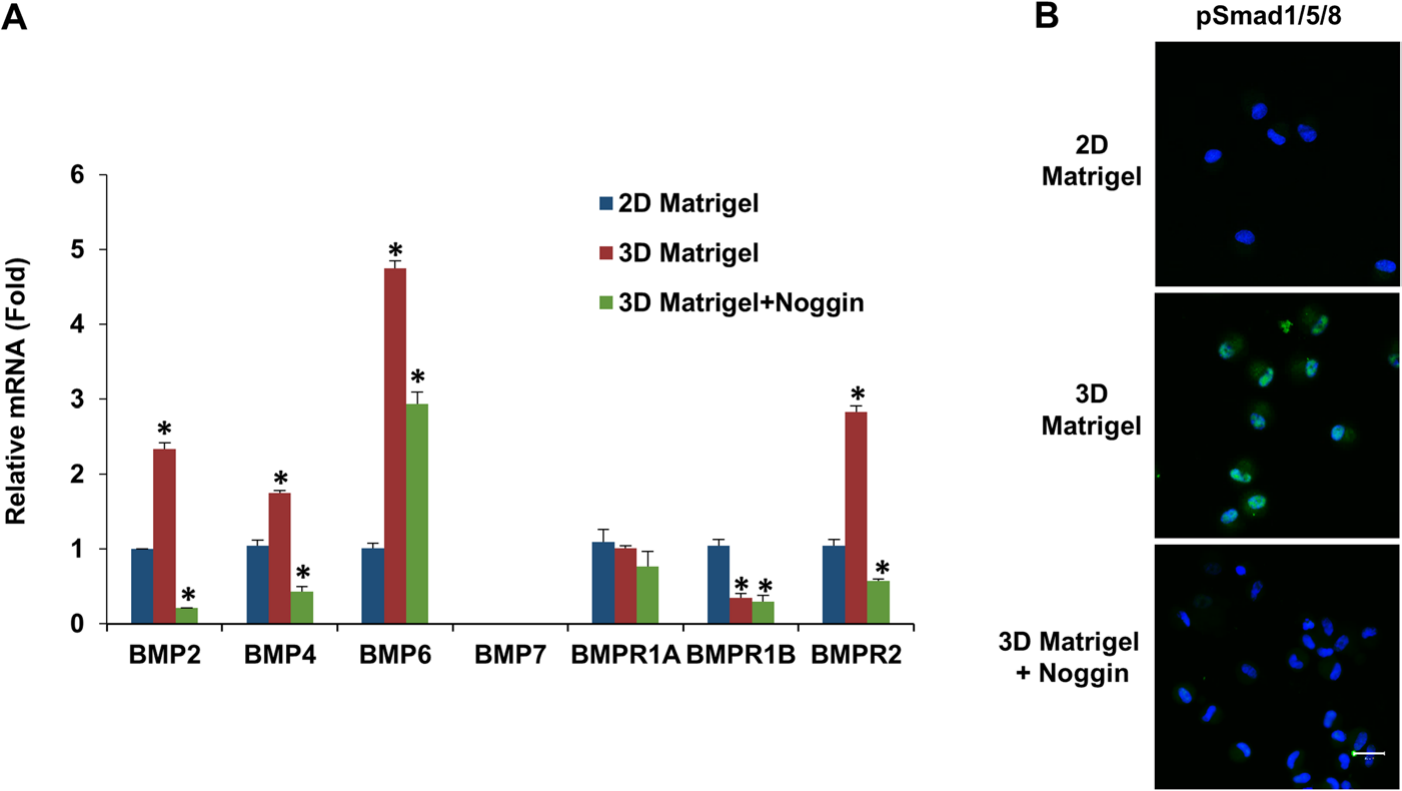
Activation of canonical BMP signaling on 3D Matrigel. P3 cells on 2D Matrigel were passaged at 2 × 10^4^/cm^2^ to 2D Matrigel as a control and to 3D Matrigel with or without Noggin in MESCM + 5% FBS before being subjected to qRT-PCR for BMP ligands and receptors using the expression level for 2D Matrigel as the control (**A**, n = 3, *P < 0.05) and immunostaining to pSmad1/5/8 (**B**, nuclear counterstaining by Hoechst 33342, scale bars: 20 µm).

Because addition of Noggin abolished the reversal effect of 3D Matrigel in upregulating expression of TM markers as well as ESC and NC markers (Fig. 4), we would like to confirm the involvement of BMP signaling, which is also involved in the reversal of the gradual phenotypic loss of human limbal niche cells by 3D Matrigel^21^. Compared to the control cultured on 2D Matrigel, TM cells exhibited notable upregulation of BMP2, BMP4, and BMP6 when reseeded on 3D Matrigel (Fig. 5A). The upregulation of BMP ligands was coupled with upregulation of BMP receptor 2 (BMPR2) but downregulation of BMPR1B (Fig. 5A). Immunostaining showed nuclear localization of pSmad1/5/8 in TM cells seeded on 3D Matrigel but not on 2D Matrigel (Fig. 5B), supporting the activation of canonical BMP signaling in the former but not the latter. Blocking of BMP signaling by Noggin abolished up-regulation of BMP2, BMP4, BMP6 and BMPR2 (Fig. 5A), blocked nuclear translocation of pSmad1/5/8 (Fig. 5B), and downregulated transcript expression of TM markers (Fig. 4A) and markers of ESCs and NCs (Fig. 4B) and immunostaining of ESC markers (Fig. 4C). These results confirmed that activation of BMP signaling was critical for maintaining the TM phenotype and progenitor status.

### Expanded TM cells are multipotent progenitors

**Figure 6:**
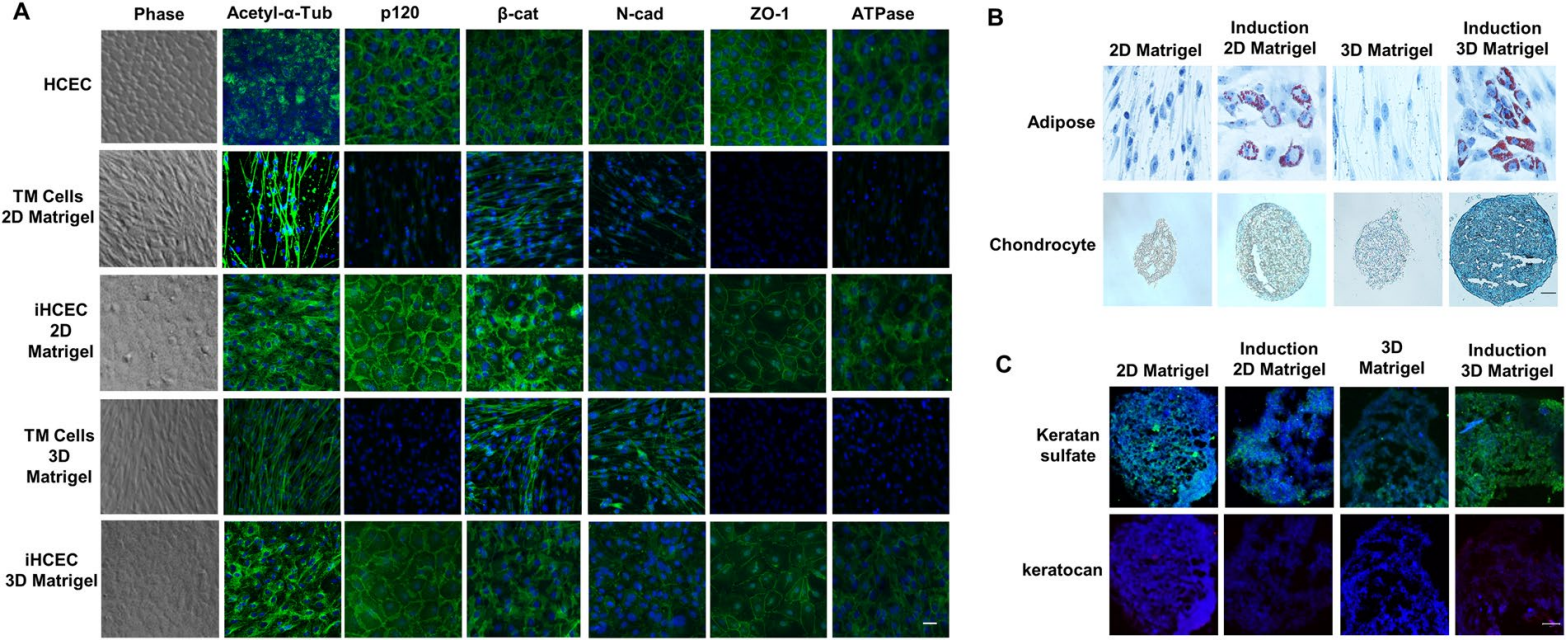
Expanded TM cells are multipotent progenitors. Using P3 cells cultured on 2D Matrigel as a control, P3 cells reseeded in 3D Matrigel were subjected to a number of differentiation assays. TM cells cultured on 2D Matrigel and in 3D Matrigel could be induced into HCEC-like cells (**A**) and adipose cells (**B**). However, TM cells cultured in 3D Matrigel could but those on 2D Matrigel could not be induced to chondrocytes (**B**). Both cells could not be induced to keratocytes (**C**). Nuclear counterstaining by Hoechst 33342 (**A**,**C**). Scale bars: 50 µm (**B**) and 20 µm (**A**,**C**).

Because expanded TM cells also express markers of ESC and NC (Figs 1 and 4), we wondered whether they were multipotent. Using P3 cells cultured on 2D Matrigel as a control, we subjected them together with P2 cells reseeded on 3D Matrigel to a number of differentiation assays achieved by respective differentiation media. For comparison, normal human corneal endothelial cells (HCEC) were cultured and formed a monolayer of hexagonal cells expressing a typical immunostaining pattern for several markers such as acetyl-α-tubulin (ciliary pattern), p120-catenin (p120), β-catenin, N-cadherin, Zona occludens protein 1 (ZO-1) and Na^+^/K^+^-ATPase (all intercellular junctions) (Fig. 6A, top panel)^22,23^. Before induction, TM cells on both 2D Matrigel and 3D Matrigel were spindle shaped (Fig. 6A, Phase) and did not exhibit any of the aforementioned HCEC staining pattern (Fig. 6A). However, after induction, TM cells on both 2D Matrigel and 3D Matrigel turned into more hexagonal and expressed an immunostaining pattern similar to HCEC with positive expression of p120, β-catenin, N-cadherin, ZO-1, and Na^+^/K^+^-ATPase preferentially in intercellular junctions (Fig. 6A). The staining for acetyl-α-tubulin was also peri-membranous as contrasted to the central punctate staining at the center of the hexagon as reported in HCEC^22–25^. All cells did not show any positive staining to LEF1 and S100A4, which are markers of endothelium-mesenchymal transition^22^ (not shown), indicating that these cells were not transitioned to a pathological state. These results suggested that TM cells expanded on 2D Matrigel or 3D Matrigel still retained the plasticity to be differentiated into HCEC-like cells. In addition, TM cells expanded on both 2D and 3D Matrigel could be differentiated into adipocytes (Fig. 6B). Aggregates of TM cells cultured on 3D Matrigel could, but those cultured on 2D Matrigel could not be induced to chondrocytes as evidenced by Alcian Blue staining (Fig. 6B), which was confirmed by measuring OD at 450 nm (not shown)^18^. In contrast, TM cells on both 2D and 3D Matrigel could not be induced into keratocytes as shown by negative staining of keratocan (Fig. 6C), which is corneal stromal specific extracellular matrix^8^ and could not be induced into osteocytes (not shown).

## Discussion

Human trabecular meshwork cells are endothelial-like cells in a connective tissue located close to the front of the eye. However, most of authors described the cell shape as “spindle” when the cells were cultured *in vitro*^26–28^. We have found that the morphology of TM primary culture was indeed spindle when scattered (Fig. 1). We have also noted that TM cells would be spindle after confluence when cultured on different substrates except 2D Matrigel plus MESCM-based media (Fig. 1), suggesting that TM cells can keep their *in vivo* phenotype when cultured on 2D Matrigel in MESCM-based media.

TM cells characteristically express Vim (a stromal cell marker), aquaporin-1 (AQP-1), matrix gla protein (MGP) and chitinase 3-like 1 (CHI3L1) and AnkG^29–34^. Interestingly, there is also a population of progenitors in Schwalbe’s Ring, the translation area between the periphery of corneal endothelium and the anterior non-filtering portion of TM, evidenced to have the capability of forming free-floating neurospheres, a function associated with multipotent progenitors^12^ expressing Oct-3/4, nestin, and Sox2^7,8^. It is unclear how the entire population of TM cells can be best expanded with both TM and progenitor characteristics without losing cell phenotype in various *in vitro* culture conditions such as cell isolation methods, culture substrates and culture media.

Previously, the substrates such as gelatin (collagen)^10,11,13^ and fibronectin^8,9^ have been used to culture TM cells. Herein, our comparative analysis disclosed that 2D Matrigel, i.e., a solubilized basement membrane matrix containing laminin, collagen IV, heparin sulfate proteoglycans, entactin/nidogen, and a number of growth factors (for review, see ref.^35^), was better than collagen IV and fibronectin in order to maintain TM as cuboidal cells and express the normal phenotype of TM cells based on the TM markers such as AQP1, MGP, CHI3L1, and AnkG (Fig. 1). This result resembles our success in using 2D Matrigel to isolate and expand human limbal niche progenitors^16–18,20,36^ and suggests that the *in vivo* extracellular matrix components such as laminins, collagens, elastin, fibronectin, fibrillins, proteoglycans, and matricellular proteins (reviewed in ref.^37^) collectively play an important role in the maintenance of TM phenotype. Unlike human limbal niche progenitors where *in vitro* expansion can be achieved in serum-free MESCM^16–18,20^, successful expansion of TM cells on 2D Matrigel requires addition of 5% FBS (Figs 2 and S1). We chose 2D culture for expansion and 3D culture for restoration of the normal phenotype based on our prior work on human limbal niche cells^16–18,20^. As noted in our prior work, direct seeding cells on 3D Matrigel maintains the normal phenotype but cells cease proliferation, thus resulting in the failure of *ex vivo* expansion. Similar to human limbal niche cells, TM cells cannot be expanded when seeded directly on 3D Matrigel. The underlying reason for 2D Matrigel to help expand TM cells or limbal niche cells remains unknown. We speculate that this might have something to do with the matrix rigidity, which influence the cell shape. Although the exact mechanism remains unknown to explain the necessity of FBS to expand TM cells, others have always included serum in their culturing of TM cells^10–15^.

Interestingly, such a population of TM cells also express ESC markers such as KLF4, Oct4, Sox2, Nanog, CD73, CD166 and Bmi1 but not CD90 and NC markers such as p75NTR, Sox9, FOXD3 (Figs 1, 3 and 4, Supplemental Fig. 2). The discrepancy for expression of CD90 from Du *et al*.^8^ is probably due to difference of the culture system, for example, different culture substrates and media. In addition, the cells form spheres when reseeded in 3D Matrigel after digestion in MESCM (Fig. 4). These phenomena resemble the characteristics of induced pluripotent stem cells (iPSCs) expressing Oct4, Sox2, KLF4 and Myc (OSKM)^38^, and limbal niche progenitors expressing embryonic stem cells and neural crest markers such as Oct4, Sox2, SSEA4, and Nanog, Nestin, N-Cadherin, and CD34^16,18,39^, suggesting this population of the cells are a group of the cells with adult progenitor characteristics. Our results support the conclusion that there is a population of TM progenitors in the trabecular meshwork^6^, and such a population of the progenitors not only express ESC and NC but also TM markers.

However on 2D Matrigel, like human limbal niche cells^20,39^, expanded TM cells also gradually lost expression of TM markers and ESC and NC markers (Fig. 3) and such a loss was transient because it could be regained by seeding P3 cells on 3D Matrigel in MESCM + 5% FBS to yield sphere growth (Fig. 4). Interestingly, the expression of CD73, CD166 and Bmi1 is not affected by 3D Matrigel (Supplemental Fig. 2). We also attribute this difference from Du *et al*.^8^ to the different culture systems. These results collectively support the use of this *in vitro* culturing model system to expand TM cells. In 3D Matrigel, also like human limbal niche progenitors^40^, the recovery of TM phenotype as progenitor status depends on activation of canonical BMP signaling (Fig. 5) as evidenced by the nullifying effect by BMP inhibitor Noggin (Figs 4 and 5). Our study showed that 3D Matrigel activates canonical BMP signaling (not only promotes expression of BMP2, BMP4 and BMP6, but also inhibits expression of BMPR1B and BMPR2, and promotes nuclear accumulation of pSMAD1/5/8 (Fig. 5). This finding of activation of BMP canonical signaling is also reported in human limbal niche cells, of which the culturing system does not include FBS^21^. This finding strongly suggests that activation of BMP signaling is not due to the inclusion of FBS, which contains multiple growth factors and cytokines.

Expanded human limbal niche progenitor cells in 3D Matrigel have the potential of differentiation into angiogenesis/pericyte progenitors^17^ and exhibit a better potential than human bone marrow MSCs to differentiate into adipocytes, chondrocytes, and osteocytes^18^. Although P3 human TM cells expanded on 3D Matrigel also showed overexpression of ESC and NC markers (Fig. 4) [similar to human limbal niche progenitors^17^ although not tested for angiogenesis/pericyte differentiation], they exhibited the potential to differentiate into adipocytes and chondrocytes but not osteocytes (Fig. 6). P3 TM cells cultured on 2D or 3D Matrigel could be differentiated into adipocytes, however only P3 TM cells cultured on 3D Matrigel could be differentiated into chondrocytes (Fig. 6). Du *et al*.^8^ reported that their expanded human TM cells can be differentiated into keratocyte-like cells. Herein, we noted that our expanded TM cells were not able to differentiate into keratocyte-like cells due to negative staining of keratocan but could be differentiated into human corneal endothelium-like cells as assessed by the morphology and expression of HCEC markers (Fig. 6) that have been reported by us^22,23^. The discrepancy between our finding and that by Du *et al*.’s work in the lack of keratocyte differentiation might also be owing to the use of a different expansion medium. Collectively, these results strongly suggest that we have developed a novel *in vitro* model system to expand TM progenitors that have the potential of being differentiated into different NC lineages. That is, we have optimized the method of expanding TM progenitor cells using the unique combination of collagenase A (reported previously by Stamer *et al*.^10^) and 2D Matrigel + MESCM + 5% FBS for effective isolation and expansion of TM cells and 3D Matrigel for phenotype reversion of the expanded TM cells to that *in vivo*. Using this method, we are able to expand the small cuboidal TM progenitors expressing markers of TM, ESC, and NC cells effectively. Such expanded TM progenitors may be used to study pathogenesis of glaucoma and corneal endothelial dysfunction and may also have the potential to be deployed as a regenerative therapy for treating these blinding diseases.

## Methods

### Materials

Materials used for isolation, culture and relevant experiments of TM cells were listed in Supplementary Table S4. Assay IDs for Real-time PCR primers were presented in Supplementary Table S5. Sources of antibodies used for immunofluorescence Staining were shown in Supplementary Table S6.

### Isolation and Expansion of Human Corneal Endothelial Cells and TM Cells

This research protocol was approved by Institutional Review Board of Tissue Tech Inc., Miami, Florida. Thirty-two corneoscleral rims from 18 to 60 years old donors were obtained from the Florida Lions Eye Bank (Miami, FL) and managed in accordance with the Declaration of Helsinki. The identities of these anonymous cadaver donors could not be identified. In brief, human corneal endothelial cells (HCEC) were isolated as reported^41–43^. Under a dissecting microscope, the corneoscleral rim was stripped off the adherent iris and was cut through the inner edge of Schwalbe’s line. After rinsing three times with MESCM, the Descemet’s membrane was stripped and digested at 37 °C for 16 h with 2 mg/ml collagenase A in MESCM + 5%FBS. The resultant HCEC aggregates were collected by centrifugation at 1,000 rpm for 5 min to remove the digestion solution and cultured in 24-well dishes coated with collagen IV in MESCM, which was made of DMEM/F-12 (1:1) supplemented with 10% knockout serum, 5 µg/ml insulin, 5 µg/ml transferrin, 5 ng/ml sodium selenite, 4 ng/ml Fibroblast growth factor-basic (bFGF), 10 ng/ml human leukemia inhibitory factor (hLIF), 50 µg/ml gentamicin, and 1.25 µg/ml amphotericin. TM cells were isolated according to Du *et al*.^8^ and Stamer *et al*.^10^ with modifications. The remaining corneoscleral rim, after a cut made at the Schwalbe’s line, TM tissue was removed and digested with collagenase A in MESCM + 5% FBS at 37 °C for 16 h. The resultant cells were washed once in MESCM + 5% FBS and seeded at a density of 2 × 10^3^ per cm^2^ on plastic or plastic coated with 2D Matrigel by adding 5% diluted Matrigel^16^ after 1 h incubation at 37 °C, or coated with fibronectin (<0.01% fibronectin) or collagen IV (10 µg/ml) after overnight incubation at 4 °C in MESCM + 5% FBS, MESCM, or SCGM which is made of OptiMEM-1 supplemented with 5% fetal bovine serum, 10 ng/mL epidermal growth factor (EGF), 100 µg/mL bovine pituitary extract, 20 µg/mL ascorbic acid, 200 µg/mL calcium chloride, 0.08% chondroitin sulfate, 100 IU/mL penicillin, 100 µg/mL streptomycin, and 50 µg/mL gentamicin^8,9^. Upon 80–90% confluence, cells on 2D Matrigel in MESCM or MESCM + 5%FBS were passaged serially at a density of 5 × 10^3^ per cm^2^. The extent of total expansion was measured by the number of population doubling using the following formula: number of cell doublings (NCD) = log_10_(y/x)/log_10_2, where y is the final density of the cells and x is the initial seeding density of the cells^16^.

### Maintenance of TM Progenitor Cells

P2 TM cells cultured on 2D Matrigel in MESCM + 5% FBS were passaged at the density of 5 × 10^3^ cells per cm^2^ on 2D Matrigel in MESCM + 5% FBS for 1 week or at the density of 1 × 10^5^ cells per cm^2^ on 3D Matrigel that was made by adding 50% diluted Matrigel^16^ in MESCM + 5% FBS for 48 h. The resultant P3 cells on either 2D or 3D Matrigel were then harvested for the following experiments. Spheres formed by TM cells on 3D Matrigel were isolated by digestion with 10 mg/ml dispase II at 37 °C for 2 h and rendered into single cells by 0.05% trypsin and 1 mM EDTA before being examined for their multipotent potential via various differentiation assays (see below) when compared to P3 cells cultured on 2D Matrigel. In addition, Noggin, a BMP signaling inhibitor, was also added at the final concentration of 500 ng/ml for 48 h in sphere cultures on 3D Matrigel^40^.

### Differentiation Assays

For differentiation into keratocytes, 3 × 10^5^ cells were spun down for pellet preparation and cultured in the Keratocyte Differentiation Medium consisting of Advanced DMEM with 10 ng/ml fibroblast growth factor 2 and 0.5 mM ascorbic acid; Thermo Fisher Scientific) for 21 days^44^. For differentiation into corneal endothelial cells, single cells were seeded at the density of 1 × 10^4^ cells per cm^2^ in 24-well plate coated with collagen IV in MESCM + 5% FBS. After cell adhesion in 24 h, the medium was switched to Corneal Endothelial Differentiation Medium consisting of low-glucose DMEM with 10% FBS and 50 µg/ml gentamicin, and 1.25 µg/ml amphotericin B) for 28 days^45^. For differentiation into adipocytes or osteocytes, single cells were seeded at the density of 1 × 10^4^ cells per cm^2^ in 24-well plate coated with Matrigel in MESCM + 5% FBS. After cell adhesion in 24 h, the medium was switched to the Adipogenesis Differentiation Medium (Invitrogen) or Osteogenesis Differentiation Medium (Invitrogen), respectively, for 21 days^18^. For differentiation into chondrocytes, 3 × 10^5^ cells were prepared for pellets and cultured in Chondrogenesis Differentiation Medium (Life Line) for 28 days^18^. All the above media were changed every 3 days.

### RNA Extraction, Reverse Transcription, and Real-time PCR

Total RNAs were extracted using an RNeasy Mini kit (QIAGEN) and reverse-transcribed using a High Capacity Reverse Transcription kit (Life Technologies). cDNA of each cell component was amplified by RT-real-time PCR using specific primer–probe mixtures and DNA polymerase in a real-time PCR system (7000; Life Technologies). The real-time RT-PCR profile consisted of 10 min of initial activation at 95 °C, followed by 40 cycles of 15 sec denaturation at 95 °C, and 1 min annealing and extension at 60 °C. All TagMan Gene Expression Assays are listed in Supplementary Table S2.

### Immunofluorescence, Oil Red O and Alcian Blue staining

For characterization of induction of endothelium, immunostaining of corneal endothelial markers such as acetyl-α-tubulin, p120, Na^+^/K^+^-ATPase, ZO-1, N-cadherin and β-catenin and endothelial-mesenchymal transition (EMT) markers such as LEF1 and S100A4 was performed as described^23–25,43^. For characterization of induction of keratocytes, immunostaining of keratocyte markers keratan sulfate and keratocan was performed as reported^18^. In brief, single cells were prepared by cytospin after trypsin/EDTA digestion and centrifuge at 1000 rpm for 8 min (StatSpin, Inc., Norwood, MA), fixed with 4% paraformaldehyde for 15 min, permeabilized with 0.2% Triton X-100 in PBS for 30 min, and blocked with 2% BSA in PBS for 1 h before being incubated with specific primary antibodies overnight at 4 °C. After washing with PBS, samples were incubated with corresponding secondary antibodies for 1 h in room temperature using appropriate isotype-matched nonspecific IgG antibodies as controls. The nucleus was counterstained with Hoechst 33342 before being analyzed with a Zeiss LSM 700 confocal microscope (LSM700; Carl Zeiss, Thornhood, NY). For characterization of induced adipocytes, the samples were stained with Oil Red O. Oil Red O-positive cells were counted in a total of 2000 cells in triplicates as reported^18^. For characterization of induced chondrocytes, the samples were stained with Alcian Blue and quantified following the manufacturer’s protocol by measuring OD at 450 nm^18^.

### Statistical Analysis

All summary data were reported as means ± SD calculated for each group and compared using ANOVA and the Student’s paired and unpaired t test using Excel software (Microsoft). Test results were reported as two-tailed p-values, where p < 0.05 was considered statistically significant.

## Electronic supplementary material

Supplementary Information

## References

[1] Alvarado J, Murphy C, Polansky J, Juster R (1981). Age-related changes in trabecular meshwork cellularity. Investigative ophthalmology & visual science.

[2] He Y (2008). Mitochondrial complex I defect induces ROS release and degeneration in trabecular meshwork cells of POAG patients: protection by antioxidants. Investigative ophthalmology & visual science.

[3] Lutjen-Drecoll E (2005). Morphological changes in glaucomatous eyes and the role of TGFbeta2 for the pathogenesis of the disease. Exp Eye Res.

[4] Le A, Mukesh BN, McCarty CA, Taylor HR (2003). Risk factors associated with the incidence of open-angle glaucoma: the visual impairment project. Investigative ophthalmology & visual science.

[5] Braunger BM (2014). Identification of adult stem cells in Schwalbe’s line region of the primate eye. Investigative ophthalmology & visual science.

[6] Acott TS (1989). Trabecular repopulation by anterior trabecular meshwork cells after laser trabeculoplasty. Am J Ophthalmol.

[7] McGowan SL, Edelhauser HF, Pfister RR, Whikehart DR (2007). Stem cell markers in the human posterior limbus and corneal endothelium of unwounded and wounded corneas. Mol.Vis..

[8] Du Y (2012). Multipotent stem cells from trabecular meshwork become phagocytic TM cells. Investigative ophthalmology & visual science.

[9] Du Y, Yun H, Yang E, Schuman JS (2013). Stem cells from trabecular meshwork home to TM tissue *in vivo*. Investigative ophthalmology & visual science.

[10] Stamer WD, Seftor RE, Williams SK, Samaha HA, Snyder RW (1995). Isolation and culture of human trabecular meshwork cells by extracellular matrix digestion. Curr Eye Res.

[11] Stamer DW, Roberts BC, Epstein DL, Allingham RR (2000). Isolation of primary open-angle glaucomatous trabecular meshwork cells from whole eye tissue. Curr Eye Res.

[12] Gonzalez P, Epstein DL, Luna C, Liton PB (2006). Characterization of free-floating spheres from human trabecular meshwork (HTM) cell culture *in vitro*. Exp Eye Res.

[13] Xue W, Comes N, Borras T (2007). Presence of an established calcification marker in trabecular meshwork tissue of glaucoma donors. Investigative ophthalmology & visual science.

[14] Yu AL, Fuchshofer R, Kampik A, Welge-Lussen U (2008). Effects of oxidative stress in trabecular meshwork cells are reduced by prostaglandin analogues. Investigative ophthalmology & visual science.

[15] Goel M, Sienkiewicz AE, Picciani R, Lee RK, Bhattacharya SK (2011). Cochlin induced TREK-1 co-expression and annexin A2 secretion: role in trabecular meshwork cell elongation and motility. PLoS One.

[16] Xie HT, Chen SY, Li GG, Tseng SC (2012). Isolation and expansion of human limbal stromal niche cells. Investigative ophthalmology & visual science.

[17] Li GG, Chen SY, Xie HT, Zhu YT, Tseng SC (2012). Angiogenesis potential of human limbal stromal niche cells. Investigative ophthalmology & visual science.

[18] Li GG, Zhu YT, Xie HT, Chen SY, Tseng SC (2012). Mesenchymal stem cells derived from human limbal niche cells. Investigative ophthalmology & visual science.

[19] Kelley MJ (2009). Stem cells in the trabecular meshwork: present and future promises. Exp Eye Res.

[20] Xie HT, Chen SY, Li GG, Tseng SC (2011). Limbal epithelial stem/progenitor cells attract stromal niche cells by SDF-1/CXCR4 signaling to prevent differentiation. Stem Cells.

[21] Chen SY (2015). HC-HA/PTX3 Purified from Amniotic Membrane Promotes BMP Signaling in Limbal Niche Cells to Maintain Quiescence of Limbal Epithelial Progenitor/Stem Cells. Stem Cells.

[22] Zhu YT, Chen HC, Chen SY, Tseng SC (2012). Nuclear p120 catenin unlocks mitotic block of contact-inhibited human corneal endothelial monolayers without disrupting adherent junctions. J.Cell Sci..

[23] Zhu, Y. T. *et al*. Activation of RhoA-ROCK-BMP signaling reprograms adult human corneal endothelial cells. *J*.*Cell Biol*. (2014). 10.1083/jcb.201404032 PMC4164941 25202030

[24] Liu Y (2017). Human Corneal Endothelial Cells Expanded *In Vitro* Are a Powerful Resource for Tissue Engineering. Int J Med Sci.

[25] Liu Y (2017). Characterization and Prospective of Human Corneal Endothelial Progenitors. Int J Med Sci.

[26] Lin S, Lee OT, Minasi P, Wong J (2007). Isolation, culture, and characterization of human fetal trabecular meshwork cells. Curr Eye Res.

[27] Stamer WD, Clark AF (2017). The many faces of the trabecular meshwork cell. Exp Eye Res.

[28] Nadri S (2013). Mesenchymal stem cells from trabecular meshwork become photoreceptor-like cells on amniotic membrane. Neuroscience letters.

[29] Foets B, van den Oord J, Engelmann K, Missotten L (1992). A comparative immunohistochemical study of human corneotrabecular tissue. Graefe’s archive for clinical and experimental ophthalmology=Albrecht von Graefes Archiv fur klinische und experimentelle Ophthalmologie.

[30] Stamer WD, Seftor RE, Snyder RW, Regan JW (1995). Cultured human trabecular meshwork cells express aquaporin-1 water channels. Curr Eye Res.

[31] Liton PB (2005). Specific targeting of gene expression to a subset of human trabecular meshwork cells using the chitinase 3-like 1 promoter. Investigative ophthalmology & visual science.

[32] Gipson IK, Anderson RA (1979). Actin filaments in cells of human trabecular meshwork and Schlemm’s canal. Investigative ophthalmology & visual science.

[33] Chang IL (1991). Expression of modified low-density lipoprotein receptors by trabecular meshwork cells. Curr Eye Res.

[34] Stamer WD (1996). Cultured human trabecular meshwork cells express functional alpha 2A adrenergic receptors. Investigative ophthalmology & visual science.

[35] Hughes CS, Postovit LM, Lajoie GA (2010). Matrigel: a complex protein mixture required for optimal growth of cell culture. Proteomics.

[36] Zhang Y (2016). The Limbal Epithelial Progenitors in the Limbal Niche Environment. Int J Med Sci.

[37] Abu-Hassan, D. W., Acott, T. S. & Kelley, M. J. The Trabecular Meshwork: A Basic Review of Form and Function. *J Ocul Biol* **2** (2014). 10.13188/2334-2838.1000017 PMC4209746 25356439

[38] Takahashi K, Yamanaka S (2006). Induction of pluripotent stem cells from mouse embryonic and adult fibroblast cultures by defined factors. Cell.

[39] Chen SY, Hayashida Y, Chen MY, Xie HT, Tseng SC (2011). A new isolation method of human limbal progenitor cells by maintaining close association with their niche cells. Tissue Eng Part C Methods.

[40] Han B, Chen SY, Zhu YT, Tseng SC (2014). Integration of BMP/Wnt signaling to control clonal growth of limbal epithelial progenitor cells by niche cells. Stem Cell Res..

[41] Li W (2007). A novel method of isolation, preservation, and expansion of human corneal endothelial cells. Invest Ophthalmol.Vis.Sci..

[42] Zhu YT (2008). Characterization and comparison of intercellular adherent junctions expressed by human corneal endothelial cells *in vivo* and *in vitro*. Invest Ophthalmol.Vis.Sci..

[43] Lu WJ (2016). Senescence Mediated by p16INK4a Impedes Reprogramming of Human Corneal Endothelial Cells into Neural Crest Progenitors. Sci Rep.

[44] Du Y (2007). *Secretion and organization of a cornea-like tis*sue *in vitro* by stem cells from human corneal stroma. Investigative ophthalmology & visual science.

[45] Hara, S. *et al*. Identification and Potential Application of Human Corneal Endothelial Progenitor Cells. *Stem Cells Dev*. (2014). 10.1089/scd.2013.0387 24588720

