# Supplementary material for: Isolation and Expansion of Multipotent Progenitors from Human Trabecular Meshwork
Source: Sci Rep. 2018 Feb 12;8:2814. doi: 10.1038/s41598-018-21098-2 (PMC5809375; doi:10.1038/s41598-018-21098-2)
Supplement: Supplementary file 1 — Supplementary Information [file 41598_2018_21098_MOESM1_ESM.pdf]

Isolation and Expansion of Multipotent Progenitors from Human Trabecular Meshwork

Yuan Zhang[1*], Subo Cai[2*], Scheffer C. G. Tseng[1] and Ying-Ting Zhu[1#]

[1]Research and Development Department, Tissue Tech, Inc., Miami, FL, USA

[2]Department of Ophthalmology, Union Hospital, Tongji Medical College, Huazhong University of Science and Technology, Wuhan, China

[*]The first two authors contribute equally to this manuscript.

Short Running Head: Multipotent Progenitors from Trabecular Meshwork

Key Words: isolation, expansion, trabecular meshwork, progenitors, corneal endothelium, BMP

[#]**Author for Correspondence**: Ying-Ting Zhu, Ph.D., R&D Department, TissueTech, Inc., 7000 SW 97th Avenue, Suite 212, Miami, FL 33173. Telephone: (786) 456-7632; Fax: (305) 274-1297; E-mail: yzhu@tissuetechinc.com

Supplementary Information

Supplementary Fig. S1. Morphological analysis by phase-contrast microscopy.

TM cells were expanded on 2D Matrigel in MESCM or MESCM+5% FBS and each passage was subjected to morphological analysis by phase-contrast microscopy (scale bar: 100 µm).

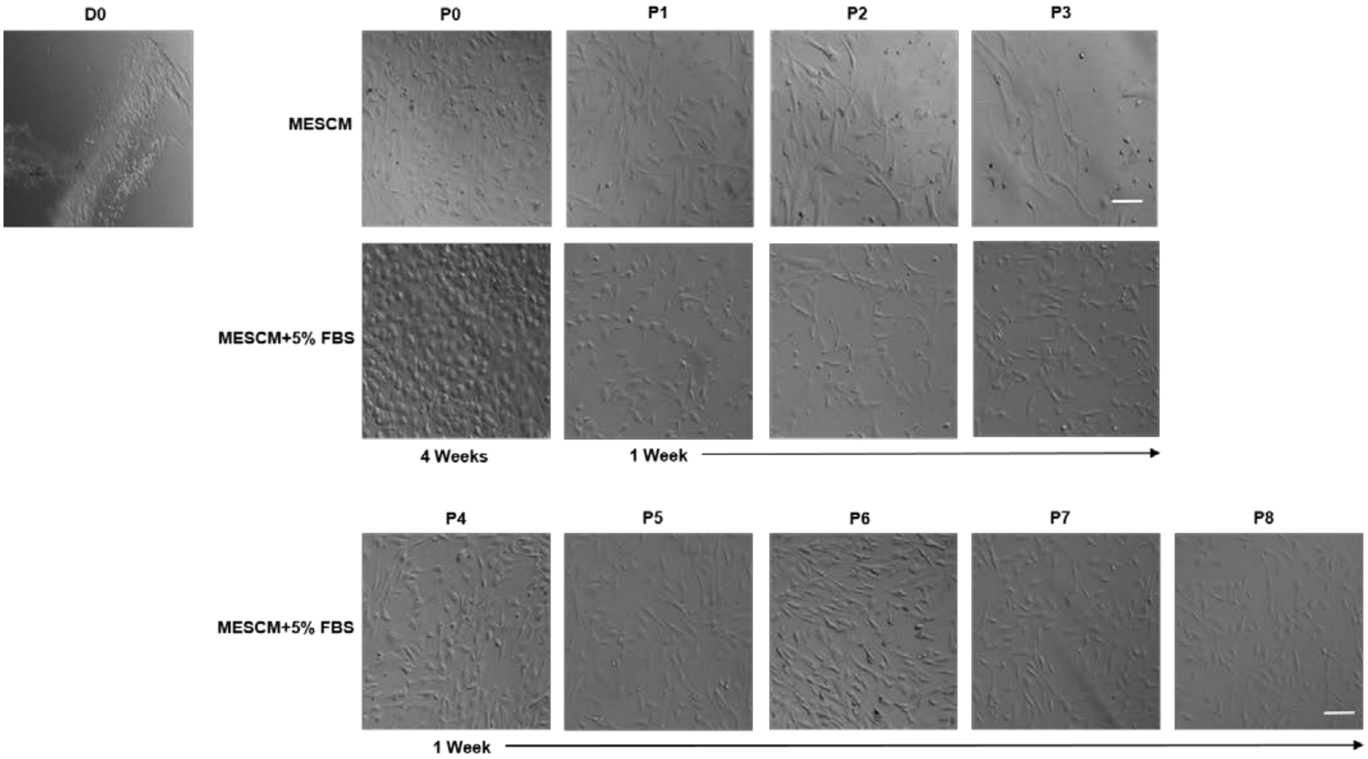

Supplementary Fig. S2. Analysis of CD73, CD90, CD166 and BMI1.

Freshly isolated TM cells (D0) and TM cells cultured on 2D Matrigel in MESCM+5% FBS were subjected to qRT-PCR for transcription expression of CD73, CD90, CD166 and BMI1 (A, using freshly isolated TM cells as the control, n=3, *P<0.05). In addition, P3 cells cultured on 2D Matrigel in MESCM+5% FBS were reseeded in 3D Matrigel with or without Noggin before analysis by qRT-PCR for CD73, CD90, CD166 and BMI1 (B, n=3, *P<0.05) by setting the expression level for 2D Matrigel as the control.

Although CD73, CD90, CD166 and Bmi1 was reported by others as TM ESC markers[1], we have noted that the expression of CD90 was not found in TM progenitor progenitors in our system. In addition, though the expression of CD73, CD166 or Bmi1 was found in TM cells expanded in our system, CD73 was upregulated from passage 1 to passage 7, CD166 was downregulated from passage 2 to passage 7, and Bmi1 was upregulated at passage 1. Furthermore, the expression of those markers were not affected by 3D Matrigel.

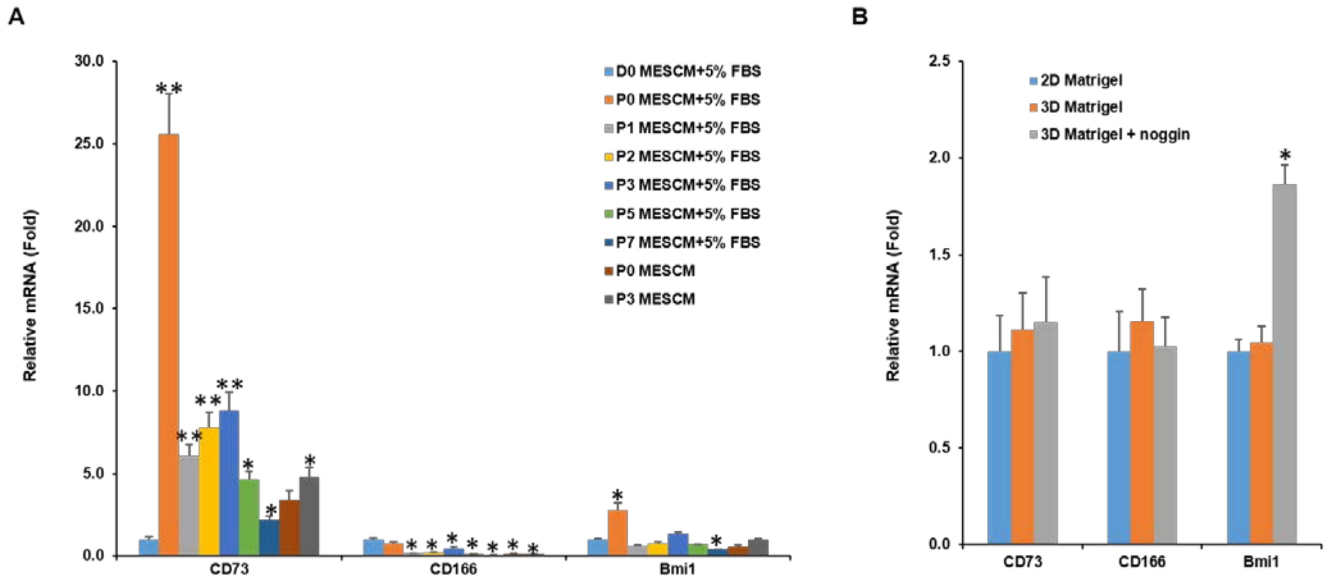

Supplementary Table S1. Data for Fig. 3A, Expanded TM cells lose their phenotype and progenitor status on 2D Matrigel

Group	Group 1	Group 2	Group 3	Group 4	Group 5	Group 6	Group 7	Group 8	Group 9	P value (compare to D0)
	D0 MESCM +5% FBS	P0 MESCM +5% FBS	P1 MESCM +5% FBS	P2 MESCM +5% FBS	P3 MESCM +5% FBS	P5 MESCM +5% FBS	P7 MESCM +5% FBS	P0 MESCM	P3 MESCM	
ABCG2	1.000 ±0.006	0.235 ±0.050	0.251 ±0.060	0.283 ±0.055	0.086 ±0.012	0.285 ±0.037	0.166 ±0.022	0.317 ±0.041	0.133 ±0.028	Group 2~9 <0.05
Myc	1.000 ±0.094	0.021 ±0.002	0.050 ±0.006	0.041 ±0.004	0.037 ±0.013	0.057 ±0.019	0.060 ±0.007	0.054 ±0.007	0.029 ±0.004	Group 2~9 <0.05
KLF4	1.000 ±0.138	0.005 ±0.0006	0.003 ±0.0006	0.005 ±0.005	0.001 ±0.00008	0.001 ±0.00009	0.001 ±0.00016	0.001 ±0.00027	0.002 ±0.00020	Group 2~9 <0.05
Nanog	1.000 ±0.109	0.100 ±0.008	0.030 ±0.003	0.036 ±0.015	0.047 ±0.006	0.003 ±0.001	0.003 ±0.0004	0.012 ±0.002	0.010 ±0.002	Group 2~9 <0.05
Nestin	1.000 ±0.103	0.083 ±0.003	0.033 ±0.003	0.075 ±0.055	0.050 ±0.007	0.021 ±0.002	0.009 ±0.001	0.013 ±0.001	0.008 ±0.001	Group 2~9 <0.05
Oct4	1.000 ±0.055	0.102 ±0.017	0.028 ±0.005	0.031 ±0.082	0.063 ±0.011	0.006 ±0.001	0.004 ±0.001	0.013 ±0.002	0.011 ±0.001	Group 2~9 <0.05
Rex1	1.000 ±0.014	0.250 ±0.003	0.061 ±0.001	0.305 ±0.065	0.243 ±0.027	0.120 ±0.017	0.143 ±0.015	0.102 ±0.012	0.057 ±0.008	Group 2~9 <0.05
Sox2	1.000 ±0.014	0.046 ±0.010	0.009 ±0.002	0.010 ±0.019	0.023 ±0.003	0.001 ±0.0001	0.002 ±0.0002	0.003 ±0.0003	0.003 ±0.0003	Group 2~9 <0.05
SSEA4	1.000 ±0.019	0.818 ±0.103	0.696 ±0.117	1.074 ±0.155	0.657 ±0.040	0.368 ±0.038	0.431 ±0.031	0.439 ±0.042	0.413 ±0.030	Group 6~9 <0.05
FOXD3	1.000 ±0.068	0.005 ±0.001	0.002 ±0.0006	0.002 ±0.001	0.001 ±0.00033	0.00005 ±0.00002	0.00008 ±0.00002	0.00019 ±0.00005	0.00026 ±0.00013	Group 2~9 <0.05
HNK1	1.000 ±0.003	0.016 ±0.003	0.004 ±0.001	0.002 ±0.0001	0.102 ±0.051	0.103 ±0.050	0.205 ±0.062	0.010 ±0.049	0.750 ±0.059	Group 2~8 <0.05
MSX1	1.000 ±0.074	0.077 ±0.012	0.044 ±0.002	0.084 ±0.008	0.028 ±0.016	0.014 ±0.001	0.011 ±0.002	0.015 ±0.002	0.004 ±0.001	Group 2~9 <0.05
p75NTR	1.000 ±0.055	0.088 ±0.003	0.006 ±0.001	0.004 ±0.001	0.093 ±0.032	0.033 ±0.011	0.089 ±0.014	0.089 ±0.014	0.013 ±0.001	Group 2~9 <0.05
Sox9	1.000 ±0.031	0.561 ±0.033	0.616 ±0.038	0.601 ±0.102	0.413 ±0.054	0.676 ±0.093	0.486 ±0.077	0.616 ±0.147	0.273 ±0.035	Group 2~5,7,9 <0.05
Sox10	1.000 ±0.209	0.567 ±0.033	0.093 ±0.034	0.022 ±0.034	0.058 ±0.040	0.0009 ±0.0003	0.0001 ±0.00001	0.111 ±0.016	0.003 ±0.0004	Group 2~9 <0.05
PDGFRβ	1.000 ±0.043	0.728 ±0.045	1.312 ±0.307	1.473 ±0.098	0.867 ±0.131	0.917 ±0.102	0.922 ±0.079	1.867 ±0.155	1.292 ±0.158	Group 8 <0.05
N-cad	1.000 ±0.013	0.161 ±0.004	0.138 ±0.001	0.133 ±0.012	0.138 ±0.010	0.037 ±0.004	0.047 ±0.003	0.067 ±0.003	0.051 ±0.004	Group 2~9 <0.05

Supplementary Table S2. Data for Fig. 3C, Expanded TM cells lose their phenotype and progenitor status on 2D Matrigel

Group	Group 1	Group 2	Group 3	Group 4	Group 5	Group 6	Group 7	Group 8	Group 9	P value (compare to D0)
	D0 MESCM +5% FBS	P0 MESCM +5% FBS	P1 MESCM +5% FBS	P2 MESCM +5% FBS	P3 MESCM +5% FBS	P5 MESCM +5% FBS	P7 MESCM +5% FBS	P0 MESCM	P3 MESCM	
Vim	1.000 ±0.030	1.060 ±0.023	0.480 ±0.090	0.380 ±0.060	0.590 ±0.009	0.193 ±0.009	0.232 ±0.018	0.289 ±0.037	0.314 ±0.013	Group 3~9 <0.05
AQP1	1.000 ±0.114	0.023 ±0.004	0.002 ±0.0004	0.003 ±0.001	0.063 ±0.002	0.056 ±0.004	0.082 ±0.003	0.180 ±0.117	0.079 ±0.013	Group 2~9 <0.05
CHI3L1	1.000 ±0.115	0.215 ±0.031	0.005 ±0.001	0.004 ±0.001	0.00025 ±0.00021	0.00008 ±0.00001	0.00001 ±0.000002	2.957 ±0.119	1.522 ±0.018	Group 2~9 <0.05
MGP	1.000 ±0.150	0.137 ±0.026	0.092 ±0.017	0.130 ±0.025	0.001 ±0.001	0.015 ±0.004	0.004 ±0.001	0.187 ±0.054	0.510 ±0.149	Group 2~9 <0.05
AnkG	1.000 ±0.130	0.041 ±0.003	0.041 ±0.005	0.067 ±0.005	0.007 ±0.002	0.014 ±0.003	0.006 ±0.001	0.095 ±0.028	0.109 ±0.026	Group 2~9 <0.05

Supplementary Table S3. Data for Fig. 4A, 3D Matrigel upregulates expression of TM markers

Group	Group 1	Group 2	Group 3	P value (compare to 2D Matrigel)
	2D Matrigel	3D Matrigel	3D Matrigel +noggin	
Vim	1.000 ±0.163	0.721 ±0.183	1.225 ±0.231	
AQP1	1.000 ±0.195	1.677 ±0.132	0.213 ±0.167	Group 2~3 <0.05
CHI3L1	1.000 ±0.278	3.126 ±0.342	62.513 ±3.739	Group 2~3 <0.05
MGP	1.000 ±0.285	60.537 ±2.636	20.121 ±1.821	Group 2~3 <0.05
AnkG	1.000 ±0.114	2.371 ±0.348	1.351 ±0.293	Group 2 <0.05

Supplementary Table S4. Materials Used for Isolation, Culture and Relevant Experiments of TM cells

Materials	Sources	Catalog number	Concentration
Amphotericin B	Thermo Fisher Scientific	15290026	50 µg/ml
Adipogenesis Differentiation Kit	Thermo Fisher Scientific	A1007001	N/A
Ascorbic acid	Sigma-Aldrich,Inc	A1300000	0.5mM
Collagenase A	Roche, Indianapolis, IN	11088793001	2 mg/ml
Complete Chondrogenesis Medium	Lifeline cell technology	LM-0022	N/A
Collagen from human placenta	Sigma-Aldrich,Inc	C5533	1 x
Dulbecco's modified Eagle's Medium (DMEM)	Thermo Fisher Scientific	21063029	DMEM/F-12 (1:1)
Dispase II	Roche, Indianapolis, IN	04942078001	10 mg/ml
F-12 nutrient mixture (F-12)	Thermo Fisher Scientific	31765035	DMEM/F-12 (1:1)
Fetal Bovine Serum (FBS)	Thermo Fisher Scientific	10082147	5%
Fibronectin coating mix (Fibronectin)	AthenaES	0407	1 x
Fibroblast growth factor 2(FGF-2)	Sigma-Aldrich,Inc	SRP4037	10 ng/ml
Gentamycin	Thermo Fisher Scientific	15710072	1.25 µg/ml
Human Fibroblast Growth Factor-Basic (bFGF)	Thermo Fisher Scientific	RFGFB50	4 ng/ml
Insulin-Transferrin-sodium selenite media supplement (ITS)	Roche, Indianapolis, IN	10394000	5 µg/ml insulin, 5 µg/ml Transferrin, 5 ng/ml sodium selenite
Knockout Serum Replacement	Thermo Fisher Scientific	10828010	10%
Leukemia inhibitory factor (LIF)	Sigma-Aldrich,Inc	L9545	10 ng/ml

Low-glucose Dulbecco's modified Eagle's Medium (DMEM)	Thermo Fisher Scientific	10567014	N/A
Matrigel™ Basement Membrane Matrix (Matrigel)	BD Biosciences	354230	50%
Noggin	Sigma-Aldrich,Inc	H6416	500ng/ml
Osteogenesis Differentiation Kit	Thermo Fisher Scientific	A1007201	N/A
Optimal Cutting Temperature Compound	Sakura Finetek USA,Inc	4583	N/A
Phosphate-Buffered Saline pH7.4 (PBS)	Thermo Fisher Scientific	10010023	1 x
Trypsin and EDTA (T/E)	Thermo Fisher Scientific	25300054	0.05% and 1 mM

Supplementary Table S5. Assay IDs for Real-time PCR (All from Thermo Fisher Scientific)

Gene Name	Assay ID (Taqman Expression Assay)	Product Length
AQP1	Hs01028916_m1	96
AnkG	Hs00241738_m1	86
ABCG2	Hs01053796_m1	83
BMP1	HS00241807_m1	86
BMP2	Hs00154192_m1	60
BMP4	Hs03676628_s1	116
BMP6	Hs01099594_m1	108
BMP7	Hs00233476_m1	73
BMPR1A	Hs01034913_g1	94
BMPR1B	Hs00176144_m1	87
BMPR2	Hs00176148_m1	69
CHI3L1	Hs01072228_m1	62
FOXD3	Hs00255287_s1	78
GAPDH	Hs02758991_g1	93
HNK1	Hs00218629_m1	64
KLF4	Hs00358836_m1	110
Myc	Hs99999003_m1	65
MGP	Hs00969490_m1	143
MSX1	Hs00427183_m1	144
Nanog	Hs02387400_g1	109
Nestin	Hs00707120_s1	81
N-cadherin	Hs01032817_m1	74
Oct4	Hs00999634_gH	64
P75NTR	Hs00609977_m1	140

PDGFRβ	Hs01019589_m1	62
Rex1	Hs00381890_m1	109
Sox2	Hs01053049_s1	91
Sox9	Hs01001343_g1	101
Sox10	Hs00366918_m1	102
SSEA4	Hs00199480_m1	86
Vimentin (Vim)	Hs00185584_m1	73

Supplementary Table S6: Sources of Antibodies Used for Immunofluorescence Staining (IF)

Antibodies	vender	Source	Catalog number
AQP1	Santa Cruz	Mouse	Sc-20810
Acetyl-α-tub	abcam	Mouse	ab24610
Alexa Fluor 488 anti–mouse IgG	Thermo Fisher Scientific	Donkey	A21202
Alexa Fluor 555 anti–bbit IgG	Thermo Fisher Scientific	Goat	A21434
Alexa Fluor 633 Anti-Goat	Thermo Fisher Scientific	Donkey	A21082
Alizarin red S	Sigma-Aldrich,Inc	N/A	A5533
Alcian blue	Sigma-Aldrich,Inc	N/A	A3157
β-catenin	BD	Mouse	610154
c-Myc	abcam	Rabbit	ab32072
CHI3L1	R&D Systems (Minneapolis, MN)	Goat	AF2599
FoxD3	abcam	Mouse	ab107248
Hoechst 33342	Sigma-Aldrich,Inc	N/A	022M4030 V
Klf4	abcam	Rabbit	ab72543
Keratan sulfate	EMD Millipore	Mouse	MAB2022
Keratocan	Kind gift from Wiston Kao	Goat	N/A
LEF1	Abcam	Rabbit	ab53293
MGP	Santa Cruz	Mouse	Sc-81546
Mounting medium	Vector laboratories , Inc.	N/A	H-1000
Nestin	abcam	Mouse	ab22035
Nanog	abcam	Rabbit	ab80892
N-cadherin	abcam	Moues	ab19348
Na-K-ATPase	EMD Millipore	Moue	05-369

Oct4	EMD Millipore	Mouse	MAB4419
Oil red O	Sigma-Aldrich,Inc	N/A	9775
P75NTR	abcam	Rabbit	ab8874
pSmad1/5/8	Cell Signaling Technology	Rabbit	9511S
P120	Santa Cruz Biotechnology, Inc.	Rabbit	SC1101
Sox2	abcam	Mouse	ab75485
Sox9	abcam	rabbit	ab26414
S100A4	abcam	Rabbit	ab27957
Vimentin	abcam	Mouse	ab8978
ZO-1	Thermo Fisher Scientific	Rabbit	617300

Reference

1 Du, Y. *et al.* Multipotent stem cells from trabecular meshwork become phagocytic TM cells. *Investigative ophthalmology & visual science* **53**, 1566-1575, doi:10.1167/iovs.11-9134 (2012).
